# Seromolecular survey and risk factor analysis of Crimean-Congo haemorrhagic fever orthonairovirus in occupationally exposed herdsmen and unexposed febrile patients in Kwara State, Nigeria

**DOI:** 10.1371/journal.pone.0303099

**Published:** 2024-05-09

**Authors:** Oluwafemi Babatunde Daodu, Joseph Ojonugwa Shaibu, Rosemary Ajuma Audu, Daniel Oladimeji Oluwayelu

**Affiliations:** 1 Department of Veterinary Microbiology, Virology Unit, Faculty of Veterinary Medicine, University of Ilorin, Ilorin, Kwara State, Nigeria; 2 Centre for Human Virology and Genomics, Nigerian Institute of Medical Research, Yaba, Lagos State, Nigeria; 3 Department of Veterinary Microbiology, Arbovirology Unit, Faculty of Veterinary Medicine, University of Ibadan, Ibadan, Oyo State, Nigeria; 4 Centre for Control and Prevention of Zoonoses, University of Ibadan, Ibadan, Oyo State, Nigeria; University of California Riverside School of Medicine, UNITED STATES

## Abstract

Crimean-Congo haemorrhagic fever virus (CCHFV) is a globally significant tick-borne zoonotic pathogen that causes fatal haemorrhagic disease in humans. Despite constituting an ongoing public health threat, limited research exists on the presence of CCHFV among herdsmen, an occupationally exposed population that has prolonged contact with ruminants and ticks. This cross-sectional study, conducted between October 2018 and February 2020 in Kwara State, Nigeria, was aimed at assessing CCHFV seroprevalence among herdsmen and non-herdsmen febrile patients, and identifying the associated risk factors. Blood samples from herdsmen (n = 91) and febrile patients in hospitals (n = 646) were analyzed for anti-CCHFV IgG antibodies and CCHFV S-segment RNA using ELISA and RT-PCR, respectively. Results revealed a remarkably high CCHFV seroprevalence of 92.3% (84/91) among herdsmen compared to 7.1% (46/646) in febrile patients. Occupational risk factors like animal and tick contact, tick bites, and hand crushing of ticks significantly contributed to higher seroprevalence in the herdsmen (p<0.0001). Herdsmen were 156.5 times more likely (p<0.0001) to be exposed to CCHFV than febrile patients. Notably, the odds of exposure were significantly higher (OR = 191.3; p<0.0001) in herdsmen with a history of tick bites. Although CCHFV genome was not detectable in the tested sera, our findings reveal that the virus is endemic among herdsmen in Kwara State, Nigeria. CCHFV should be considered as a probable cause of febrile illness among humans in the study area. Given the nomadic lifestyle of herdsmen, further investigations into CCHF epidemiology in this neglected population are crucial. This study enhances our understanding of CCHFV dynamics and emphasizes the need for targeted interventions in at-risk communities.

## Introduction

Crimean-Congo haemorrhagic fever virus (CCHFV) is a tick-borne *Orthonairovirus* belonging to the family *Nairoviridae*. It is an enveloped virus with a tripartite, single-stranded RNA genome of negative polarity consisting of small (S), medium (M), and large (L) segments [1, 2]. It was first reported in 1944 in the Crimean Peninsula after an outbreak of severe haemorrhagic fever [3], and later among humans in Congo [4, 5]. Due to its extensive geographical distribution which parallels that of its major tick vector (*Hyalomma* spp.), CCHFV is described as the most widespread tick-borne virus that causes haemorrhagic disease [1, 6, 7]. While domesticated animals (sheep, goats, and cattle) and small mammals (rodents) can be infected, they do not show any obvious clinical signs but serve as viral reservoirs and amplifier hosts [8–10].

In humans, about 90% of CCHFV infections were reported to be either asymptomatic or cause non-specific, low-grade fever with no additional clinical signs [11]. Occasionally however, following a short (about one week) incubation period, patients develop severe and often fatal haemorrhagic disease marked by high fever, malaise, myalgia, vomiting, diarrhoea, petechial rash, hematomas, and generalized bleeding [12, 13]. The disease has a case fatality rate of 5–30% and its zoonotic transmission involves direct contact with infectious blood, tissues, secretions and products from CCHFV-infected animals, bites of infected ticks, especially *Hyalomma* spp., and tick crushing [14, 15]. Generally, CCHFV affects people working with animals in livestock farms and markets (herdsmen, milkers, livestock traders, veterinarians), abattoirs (butchers and workers involved with evisceration and processing), and those concerned with animal by-products such as meat and blood meal [8, 16, 17]. As a result of its potential to cause a public health emergency and the absence of efficacious drugs or vaccines, CCHFV was recently published as a priority pathogen in the World Health Organization (WHO) R&D Blueprint list [18].

Prompt and accurate diagnosis of CCHFV infections is crucial for effective patient treatment, better disease management outcomes and prevention of potential nosocomial infections [12]. Laboratory tests for diagnosis of CCHF in humans include immunofluorescence assay (IFA), enzyme-linked immunosorbent assay (ELISA) for virus antibody (IgG, IgM) and antigen detection, virus isolation (where biosafety level 4 containment facilities are available), as well as real-time and conventional reverse transcriptase-polymerase chain reaction (RT-PCR) [19, 20]. The serological assays are sensitive to antigenic variation but generally less impacted by genetic variation [21], while the RT-PCR-based techniques target the CCHFV nucleoprotein gene region in the S segment, which is more conserved across geographical isolates [22–24].

In Nigeria, CCHFV was first identified in domestic ruminants at a livestock market, ticks especially *Hyalomma* spp., and hedgehogs [25]. While serological evidence of the virus has been reported in domestic livestock [26–28], there is scarce information on human infections. Earlier studies [29, 30] were seroepidemiological surveys conducted on apparently healthy individuals, while the recent reports of Bukbuk et al. [31, 32] were based on samples from febrile hospital patients, including those originally collected for the laboratory diagnosis of malaria, typhoid fever or hepatitis. However, studies targeted at occupationally at-risk persons such as herdsmen who live in close proximity to domestic livestock and their ticks are non-existent. This study was designed to investigate the occurrence of CCHFV infections in occupationally exposed herdsmen and febrile hospital patients with limited contact with animals in Kwara State, north-central Nigeria.

## Materials and methods

### Ethics statement

Ethical approval for this study was obtained from the Nigerian Institute of Medical Research (Institutional Review Board) (IRB/I9/006), Kwara State Ministry of Health (MOH/KS/EU/777/185 and MOH/KS/EU/777/261) and Kwara State General Hospital, Ilorin (GHI/ADM/134/VOL.I/11). In addition, an informed consent form was explained (interpreted in local language) to all participants and written consent was returned (signed or thumb-printed). All proposed participants who did not give written consent were excluded from the study. Parental consent of minors was also sought before their inclusion in the study. Stored secondary samples of febrile patients were used for this study and there was no contact with the patients.

### Study location and population

**Fig 1:**
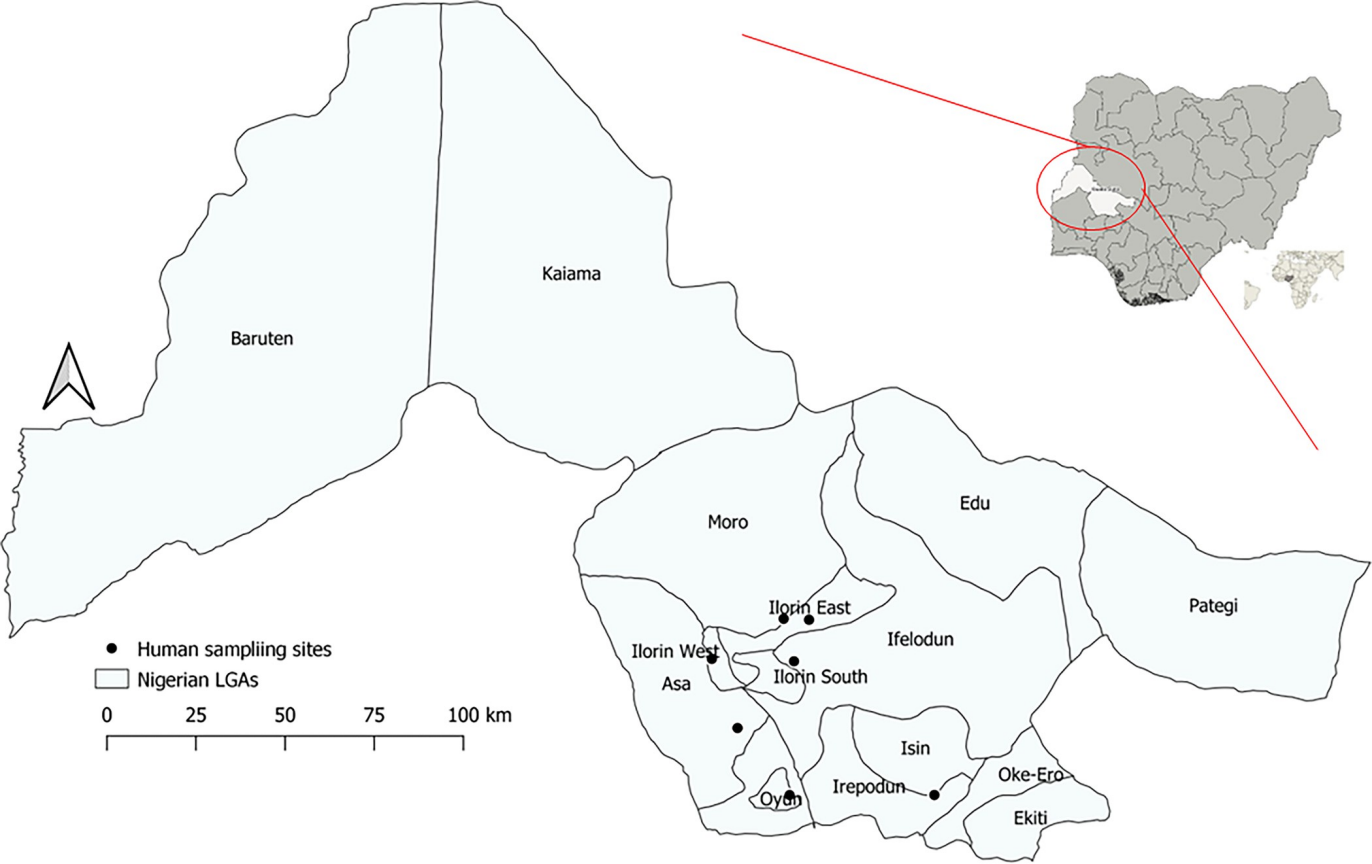
Map of Kwara State, Nigeria showing locations where study participants were recruited. The base layer of the map was created using DIVA-GIS Version 7.5 (https://www.diva-gis.org/) software.

The study was conducted at seven sampling sites (four herdsmen kraals, two secondary healthcare centres/hospitals and one tertiary/Teaching hospital) in five local government areas (LGAs) of Kwara State, namely: Ilorin West, Ilorin East, Irepodun, Asa and Offa (Fig 1). Herdsmen, especially people working with ruminants, and non-herdsmen (persons with no and or rare contact with ruminants) were the study groups.

### Study design and inclusion/exclusion criteria

This was a cross-sectional study using a stratified random sampling method. The sampling period spanned from November 5, 2017 to November 4, 2019 for the herdsmen study, while archived blood samples (with detailed information) collected from febrile patients between May 30, 2018 and April 30, 2019 were used for the non-herdsmen study. Among the herdsmen group, only persons involved in activities that bring them in direct contact with ruminants (grazing, shearing, milking) were included in the study while animal owners and others who had no direct contact with ruminants for more than six months were excluded. The non-herdsmen group included people who seldom had or did not have contact with animals but were identified as febrile patients in the selected hospitals based on high body temperatures (and were further screened for malaria parasite or typhoid fever).

### Sample collection and processing

Using sterile needles and syringes, 3 ml of blood was obtained aseptically by brachial venepuncture from each participant. The blood samples were dispensed into plain tubes and transported to the laboratory in a cold chain. Subsequently, sera were separated from the clotted blood into cryovials and spun at 3500 revolutions per minute for 10 minutes. The harvested sera were then kept at -20°C until used.

### Serology

The detection of CCHFV IgG was done using an indirect enzyme-linked immunosorbent assay (ELISA) developed and used at the National Institute for Viral Disease Control and Prevention, Chinese Center for Disease Control and Prevention, Beijing, China [33]. Briefly, wells of the microtiter plate were coated with 100 μl CCHFV nucleoprotein at 5 μg/ml in bicarbonate buffer (50 mmol/L, pH 9.6). The plates were sealed and incubated at 4°C overnight after which they were washed six times with 1X phosphate buffered saline with Tween 20 (PBS-T). Thereafter, 300 μl blocking buffer (skimmed milk) was added per well and the plate incubated for 1 hour at 37°C followed by another wash step. The test serum samples were initially diluted (1:100) in PBS in an uncoated microtiter plate before 100 μl of each was transferred into the wells of the coated plate. The plate was incubated at 37°C for 30 minutes, washed as previously described and blot-dried on paper towel. Two positive and three negative controls were included in each plate. Thereafter, 100 μl of horse-radish peroxidase-conjugated goat anti-human IgG diluted 1:1000 in PBS-T was dispensed per well, followed by incubation at 37°C for 30 minutes, as well as washing and blotting steps. Subsequently, 100 μl/well of 3’,3’,5’,5’-tetramethylbenzidine (TMB) was added and the plate incubated for 15 minutes at room temperature in the dark. The reaction was stopped by addition of 100 μl/well of 1M H_2_SO_4_ and the result read at 450 nm in a microplate ELISA reader. The test was said to be valid if the mean OD value of the positive control serum (OD_PC_) was greater than 0.35 and the ratio of the mean OD values of the positive (OD_PC_) and negative (OD_NC_) control sera was greater than 3. The percentage (S/P%) of OD_sample_/ OD_PC_ was calculated and samples with S/P% > 30% were considered positive while those with S/P% ≤ 30% were considered negative.

### RNA extraction

RNA was extracted from the sera using the Viral RNA+DNA Preparation Kit (Jena Bioscience, Germany) as recommended by the manufacturer. Briefly, 150 μl of the serum was dispensed into a 1.5 mL Eppendorf tube followed by the addition of lysis buffer (300 μl). The mixture was vortexed for 15 seconds and incubated at room temperature for 10 minutes. Binding buffer (300 μl) was then added and the mixture vortexed. The lysate was dispensed into a spin column (inserted into 2 ml collection tube) and spun for 60 seconds at 13,000 g. Afterward, the spin column was washed using Washing Buffer A (500 μl), centrifuged (13,000 g for 60 seconds) and the flow through in the collection tube discarded. This step was also repeated with Washing Buffer B (500 μl). After discarding the flow through, the spin column was placed in another collection tube and centrifuged at 13,000 g for 60 seconds to ensure total removal of the ethanol residue. Finally, the spin column was placed in a new 1.5 mL Eppendorf tube. Elution buffer (50 μl) was added and the tube incubated at room temperature for 60 seconds, after which it was spun at 13,000 g for 60 seconds to elute the RNA.

### Primer design

Specific primers for partial fragment amplification of the CCHFV S-segment gene of CCHFV Africa Clades I-III were designed using the Oligo Primer Analysis software v.7 (Table 1) and synthesized by Macrogen Europe (Amsterdam, Netherlands).

**Table 1:** Primers used for amplification of CCHFV S-segment.

Primer (5’ 3’)	Target nt length	Amplicon size (bp)	Annealing Temp. (°C)	Nucleotide sequence used (Accession no./isolate/Africa Clade
CCHFV_F1- TTACCTTGAGTGTTAGCAAAATGG	36–1396	1361	57	DQ211640.1/Senegal ArD15786/Clade I
CCHFV_R1- ATCCATGTCCTGTATGTTGAATCC				
CCHFV_F2- TGAGTGGTTCGAAAAAAATGCAGG	334–1307	974	59	DQ144418/ Congo 3010/Clade II
CCHFV_R2- TCCGAAGGTTGAGAATGGACTTGG				
CCHFV_F3- CCTATTCCTTTTGCGAGAGTGTTCC	124–1379	1256	60	KY484036.1/Nigeria UCCR4401/Clade III
CCHFV_R3- AGAGGCCACTATGTCCATGTCC				

### Nucleic acid amplification

Complementary deoxyribonucleic acid was synthesized using the SCRIPT cDNA synthesis kit (Jena Bioscience, GmBH, Germany) following the manufacturer’s instructions. DNA amplification was then carried out in a MiniAmp Plus Thermal Cycler (Applied Biosystems) using the specific primers in a 20 μl reaction mixture containing 4 μl Red Load Taq master mix (Jena Bioscience, Germany), 0.4 μl each of forward and reverse primers, 5 μl cDNA template and 10.2 μl RNase-free water. The thermal cycling conditions used were 90°C/50 seconds, followed by 35 cycles of 94°C/30 seconds, 57–60°C/30 seconds, and 72°C/45 seconds. Separate reactions were conducted for each of the primer pair. The positive control was CCHFV RNA obtained from a *Hyalomma* tick while the negative control was nuclease-free water. Amplicons were analyzed on 1.8% agarose gel using the CyFox® gel electrophoresis and live imaging device (Sysmex Partec, Germany).

### Statistical analysis

Data obtained were analyzed using Chi-square (*X*^2^) test to compare the CCHFV seroprevalence based on sociodemographic and CCHFV risk factors. Analyses were carried out with GraphPad Prism version 5.01 (San Diego, USA) and p-values < 0.05 were considered significant.

## Results

### Socio-demographic profile of participants

A total of 737 people (91 herdsmen and 646 febrile patients) participated in this study (Tables 2 and 3). Among the herdsmen, male participants (56%, n = 56) were more than the females (n = 40) while herdsmen 36–45 years old (22%, n = 20) had the highest representatives with the least from the > 65 years age group (1.1%, n = 1). The 16–55 years age range (73.6%, n = 67) represented the largest proportion of the herdsmen in this study. The herdsmen were spread across Asa, Ilorin South and Ilorin East local government areas of Kwara State, with most of them having no formal education (82.4%, n = 75) and only four (4.4%) had secondary and tertiary level education. Based on marital status, 75.8% (n = 69) of them were married while the rest were single (24.2%). Majority (83.4%, n = 85) of these herdsmen were on farm settlements while few (6.6%, n = 6) still practiced nomadism. Full-time work (83.4%, n = 85) was very popular among the herdsmen with activities such as grazing (50.5%, n = 46) and milking (42.9%, n = 39) being most common.

**Table 2:** Distribution of CCHFV IgG prevalence among herdsmen in Kwara State, Nigeria.

Features	Freq. (%)	No. positive (%)	OR (95% CI)	p-value
***Location (LGA) of Kraal***				
Asa	36 (39.6)	34 (94.4)	2.02 (0.28–18.00)	0.48
Ilorin South	27 (29.7)	25 (92.6)	1.49 (0.21–13.43)	0.70
Ilorin East	28 (30.8)	25 (89.3)	1	
***Age (years)***				
5–10	8 (8.8)	7 (87.5)	1.24 (0.11–14.02)	1.00
11–15	10 (11.0)	10 (100.0)	^a^4.20 (0.20–89.69)	0.53
16–25	14 (15.4)	14 (100.0)	^a^5.80 (0.28–121.80)	0.25
26–35	19 (20.9)	19 (100.0)	^a^7.80 (0.38–162.00)	0.23
36–45	20 (22.0)	17 (85.0)	1	
46–55	14 (15.4)	12 (85.7)	1.06 (0.15–7.34)	1.00
56–65	5 (5.5)	4 (80.0)	0.71 (0.06–8.71)	1.00
> 65	1 (1.1)	1 (100.0)	^a^1.40 (0.04–43.83)	1.00
***Gender***				
Male	51 (56.0)	47 (92.2)	0.95 (0.20–4.52)	1.00
Female	40 (44.0)	37 (92.5)	1	
***Level of Education***				
None	75 (82.4)	69 (92.0)	1	
Arabic school	12 (13.2)	11 (91.7)	0.96 (0.10–8.73)	1.00
Secondary school	2 (2.2)	2 (100.0)	^a^0.47 (0.02–10.82)	1.00
Higher National Diploma/Degree	2 (2.2)	2 (100.0)	^a^0.47 (0.02–10.82)	1.00
***Marital Status***				
Single	22 (24.2)	21 (95.5)	2.00 (0.23–17.59)	1.00
Married	69 (75.8)	63 (91.3)	1	
***Type of Facility***				
Farm settlement	85 (83.4)	78 (91.8)	^a^0.81 (0.04–15.74)	1.00
Nomadism	6 (6.6)	6 (100.0)	1	
***Employment type***				
Full-time	85 (83.4)	79 (92.9)	2.63 (0.26–26.33)	0.39
Part-time	6 (6.6)	5 (83.3)	1	
**Ac*tivit*y**				
Shearer	5 (5.5)	4 (80.0)	1	
Grazer	46 (50.5)	43 (93.5)	3.58 (0.30–43.00)	0.33
Milker	39 (42.9)	36 (92.3)	3.00 (0.25–36.13)	0.39
Driver	1 (1.1)	1 (100.0)	^a^1.00 (0.02–40.31)	1.00
**TOTAL**	**91 (100.0)**	**84 (92.3)**		

Legend: Freq.—Frequency OR—Odds Ratio 95% CI—95% Confidence interval

*Significant at p < 0.05

^a^ Odds ratio was calculated by adding 0.5 to each value

**Table 3:** Risk factors associated with CCHFV exposure among herdsmen in Kwara State, Nigeria.

Features	Freq. (%) (n = 91)	CCHFV Positive (%)	OR (95% CI)	p-value
I have been bitten by tick during this work	82 (90.1)	75 (91.5)	^a^191.30 (10.09–3625)	<0.0001*
I have used my hand/ bare foot to crush tick before	83 (91.2)	76 (91.6)	^a^ 0.60 (0.03–11.46)	1.00
I have carried young or weak animal on my shoulder while grazing before	45 (49.5)	42 (93.3)	1.33 (0.28–6.33)	1.00
I have seen ticks on my “working cloth” during or after the day’s work before	83 (91.2)	76 (91.6)	^a^0.60 (0.03–11.46)	1.00
I sleep close to my animal to prevent them from being stolen	75 (82.4)	70 (93.3)	2.00 (0.35–11.37)	0.60

Legend: Freq.—Frequency OR—Odds Ratio 95% CI—95% Confidence interval

* Significant at p < 0.05

^a^ Odds ratio was calculated by adding 0.5 to each value

Most (57.4%, n = 371) of the febrile patients visiting the hospitals were females (Table 4), while the age group with the highest number of febrile patients was between 20–29 years (31.7%, n = 205) followed by the 10–19 years and 30–39 years (22%, n = 142) age groups. Based on occupation, students recorded the highest number among the febrile patients (47.1%, n = 304), followed by traders (24.1%, n = 156).

**Table 4:** Socio-demographic profile of CCHFV IgG seropositive febrile patients in Kwara State, Nigeria.

Features	Freq.	CCHFV Positive (%)	OR (95% CI)	p-value
*Gender*				
Male	275 (42.6)	23 (8.4)	1.38 (0.76–2.52)	0.35
Female	371 (57.4)	23 (6.2)	1	
*Age (years)*				
< 5	14 (2.2)	0 (0.0)	^a^0.38 (0.02–8.45)	1.00
5–9	43 (6.7)	1(2.3)	0.32 (0.03–3.72)	0.56
10–19	142 (22.0)	15 (10.6)	1.59 (0.34–7.39)	0.74
20–29	205 (31.7)	18 (8.8)	1.30 (0.29–5.92)	1.00
30–39	142 (22.0)	7 (4.9)	0.70 (0.14–3.56)	0.65
40–49	53 (8.2)	2 (4.9)	0.53 (0.07–3.97)	0.61
50–59	29 (4.5)	2 (6.9)	1	
≥ 60	18 (2.8)	1 (5.6)	0.79 (0.07–9.45)	1.00
*Occupation*				
None	7 (1.1)	0 (0.0)	^a^0.62 (0.03–11.19)	1.000
Student	304 (47.1)	29 (9.5)	1	
Trader	156 (24.1)	11 (7.1)	0.72 (0.35–1.48)	0.48
Civil servant	83 (12.8)	5 (6.0)	0.61 (0.23–1.62)	0.39
Business	48 (7.4)	0 (0.0)	^a^0.10 (0.01–1.60)	0.02*
Teacher	11 (1.7)	1 (9.1)	0.95 (0.12–7.68)	1.00
Retiree	5 (0.8)	0 (0.0)	^a^0.85 (0.05–15.75)	1.00
Driver	20 (3.1)	0 (0.0)	^a^0.23 (0.01–3.87)	0.24
Crop farmer	12 (1.9)	0 (0.0)	^a^0.37 (0.02–6.48)	0.61
**TOTAL**	**646 (100.0)**	**46 (7.1)**		

Legend: Freq.—Frequency OR—Odds Ratio 95% CI—95% Confidence interval

* Significant at p < 0.05

^a^ Odds ratio was calculated by adding 0.5 to each value

### CCHFV serology among herdsmen

A CCHFV IgG prevalence of 92.3% (84/91) was obtained among the herdsmen (Table 2). Across the three local government areas (with four kraals) sampled, prevalence rates ranged between 89.3%-94.4%. Based on age, herdsmen 11–35 years old and those > 65 years (48.4%, 44/91) were CCHFV-seropositive while the rest had ≤ 87.5% exposure rate. There was no statistically significant difference in the CCHFV exposure rate among males (92.2%) compared to the females (92.5%). Generally, analysis of socio-demographic variables considering level of education, marital status, facility type, employment type and activity indicated that most herdsmen were exposed to CCHFV with seroprevalence ranging from 80–100%.

### CCHFV serology among febrile patients

Among febrile hospital patients, CCHFV seroprevalence was 7.1% (46/646) (Table 4). The prevalence was higher in males (8.4%, 23/275) than in females (6.2%, 23/371). Based on age group, patients aged 10–19 years had the highest seroprevalence of 10.6%, while those < 5 years old were negative (0.0%). In addition, comparison based on occupation showed that students had the highest CCHFV seroprevalence of 9.5%, followed by teachers (9.1%), traders (7.1%) and civil servant (6.0%).

### Molecular studies

None of the sera tested (91 from herdsmen and 464 from febrile patients) was positive for the target CCHFV S-segment gene, although the controls gave the expected results.

### Risk factor analysis

Among the herdsmen, 82 (90.1%) had a history of being bitten by ticks while working and of these, 91.5% (75/82) were CCHFV-seropositive (Table 3). Seropositivity based on the predominant risk factors showed that 91.2% (83/91) of the herdsmen crushed ticks with their hands, 49.5% (45/91) carried young or weak animals on their shoulders, 91.2% (83/91) noticed ticks on their clothes during and after the day’s work, while 82.4% (75/91) slept close to their animals. None of the herdsmen (100%, n = 91) had heard about CCHFV before the study.

Furthermore, the results indicated that febrile patients who had prior contact with domesticated animals (cattle, sheep, goats, chickens, dogs) (88.5%; 572/646) had CCHFV seroprevalence of 6.6% (38/572), those with history of contact with ticks (67.6%; 437/646) had 5.3% (23/437) seroprevalence, those who had been bitten by ticks (9.8%; 63/646) had 4.8% (3/63) seroprevalence and those who had used their hands to crush ticks (19.3%; 125/646) had 5.6% (7/125) seroprevalence (Table 5). There was a significant difference in CCHFV seropositivity between febrile patients who had had body contact with ticks and those who had not (p = 0.0132; OR = 0.45; 95% CI = 0.25–0.82). Overall, this study revealed that CCHFV seroprevalence was significantly higher (p < 0.0001) among herdsmen compared to febrile hospital patients when different risk factors were considered (Table 6).

**Table 5:** Risk factors associated with CCHFV exposure among febrile patients in Kwara State, Nigeria.

Features	Freq. (%) (n = 646)	CCHFV Positive (%) (n = 46)	OR (95% CI)	p-value
I have had contact with animals before	572 (88.5)	38 (6.6)	0.59 (0.26–1.31)	0.2247
I have had body contact with ticks before	437 (67.6)	23 (5.3)	0.45 (0.25–0.82)	0.0132*
I have been bitten by ticks before	63 (9.8)	3 (4.8)	0.63 (0.19–2.09)	0.6084
I have used my bare hand to crush ticks before	125 (19.3)	7 (5.6)	0.73 (0.32–1.68)	0.5637

**Key:** Freq.—Frequency OR—Odds Ratio 95% CI—95% Confidence interval

* Significant at p < 0.05

**Table 6:** Comparison of common CCHFV risk factors among herdsmen and non-herdsmen (febrile humans).

Features	Herdsmen (High risk group)		Febrile patients (Low-risk group)		OR (95% CI)	p-value
	Freq. (%) n = 91	CCHFV positive (%)	Freq. (%) n = 646	CCHFV positive (%)		
I have had contact with animals before	91 (100.0)	84 (100.0)	572 (88.5)	38 (6.6)	168.6 (72.9–390.0)	<0.0001*
I have been bitten by ticks before	82 (90.1)	75 (91.5)	63 (9.8)	3 (4.8)	214.3 (53.1–864.5)	<0.001*
I have used my bare hand to crush ticks before	83 (91.2)	76 (91.6)	125 (19.3)	7 (5.6)	183 (61.7–542.6)	<0.0001*
I have had body contact or seen ticks on my clothes before	83 (91.2)	76 (91.6)	437 (67.6)	23 (5.3)	195.4 (81.0–471.6)	<0.0001*
Total exposure (no. of sera tested)	91	84 (92.0)	646	46 (7.1)	156.5 (68.42–358.10)	<0.0001*

**Key:** Freq.—Frequency OR—Odds Ratio 95% CI—95% Confidence interval

* Significant at p < 0.05

## Discussion

Although no clinical outbreak of CCHF has been recorded in Nigeria, previous studies have provided serological and virological evidence of the disease among humans, domestic ruminants, hedgehog, or ticks [25–32]. However, little is known about the occurrence and prevalence of the disease among occupationally exposed persons in the country. According to Gordon et al. [10], knowledge on human exposure to CCHFV and the factors associated with its presence in reservoir species are crucial for better understanding of transmission dynamics and to evaluate local risks for zoonotic disease emergence. In this study, which is the first to examine CCHFV occurrence in occupationally at-risk populations in Nigeria, we investigated the presence of the virus among herdsmen in three local government areas known for large populations of cattle in Kwara State, north-central Nigeria and compared the results with those of undiagnosed febrile hospital patients in the same study area.

The high overall CCHFV seroprevalence of 92.3% obtained for herdsmen in this study s significant occupational exposure (approximately nine of every 10 herdsmen tested) and establishes endemicity of the disease among this high-risk population. Considering that the herdsmen live close to their livestock, this high seroprevalence is not unexpected as they could have contracted the virus through tick bites or common behavioral practices of the herdsmen such as hand crushing of ticks, carrying of animals on their bodies and drinking unpasteurized milk from infected animals as previously reported [8, 34, 35]. This possibility is supported by earlier reports which showed that domestic livestock (particularly cattle, sheep, and goats) are asymptomatic virus reservoirs that facilitate continued tick re-infection, putting certain occupations including herders, livestock farmers, animal handlers, abattoir/slaughter-house workers, veterinarians, and agricultural laborers at highest exposure risk in endemic areas [1, 6, 36, 37]. It is noteworthy that despite the high CCHFV seroprevalence obtained among herdsmen in this study, no outbreak of the disease in humans or animals had been reported in the study area. This could be due to poor surveillance for the disease, lack of expertise in disease recognition, or circulation of an apathogenic CCHFV strain in the region. Limitations of this study include the inability to perform a neutralization test for further confirmation of our ELISA results and the lack of access to the performance evaluation report on the assay.

In contrast, our findings revealed low (7.1%) CCHFV seroprevalence among hospital patients with undiagnosed febrile illness, all of whom had minimal contact with domesticated animals (in descending order of goats, sheep, chickens, cattle). This is noteworthy as it reveals CCHFV infection of non-occupationally exposed persons which is consistent with the report of Bukbuk et al. [32] who obtained similar results in northeastern Nigeria. Although the source of infection of the febrile patients in the present study could not be ascertained, indirect (covert) exposure to infected animals, animal products, or infected patients’ secretions could have played crucial roles. This seroprevalence is lower than 9.6% and 10.6% reported among febrile humans [29] and non-febrile humans [32] in Nigeria, respectively. This implies that very few of the febrile patients studied were exposed to CCHFV. Additionally, the finding that some of these CCHFV-positive febrile patients were also positive for *Plasmodium* parasite and *Salmonella typhi* (complete data is not available) suggests that CCHF should be included as a differential diagnosis in cases of pyrexia of unknown origin, and screening for the virus in patients should be conducted. Furthermore, the 7.1% CCHFV seroprevalence obtained in this study was higher than 2.7% and 4.2% reported among patients attending hospitals in Mozambique [38] and Greece [39], respectively, where several classical cases of CCHF had been reported. While this indicated a higher CCHFV exposure in febrile patients in Nigeria, the low to no case record of CCHF in Nigeria suggests that more still needs to be done in terms of public health awareness and the availability of rapid diagnostics at point of care as the first human CCHFV case in Nigeria was not diagnosed during routine work.

Moreover, statistical analysis indicated that the odds of being exposed to CCHFV were 156.5 times (p < 0.0001) higher in herdsmen than in febrile hospital patients. This corroborates the fact that occurrence of CCHF is largely dependent on human exposure to the virus through contact with competent tick vectors and or the viral reservoir/amplifying hosts. Specifically, herdsmen are usually in close contact with their animals, are more likely to be in frequent contact with ticks, and hence get more exposed to CCHFV and, possibly, other tick-borne viruses [40]. Moreover, a significantly higher (p = 0.02) CCHFV seropositivity was obtained for students in comparison with participants engaged in entrepreneurial activities. The reason for this observation is not known. Thus, there is a need for further investigations.

The observation that herdsmen in Asa LGA had higher CCFHV exposure than those in Ilorin East LGA could be attributed to the larger population of ruminants reared in Asa LGA compared to the latter location. In an earlier study [41], we showed that Asa LGA of Kwara State is a hot spot for Dugbe orthonairovirus, a haemorrhagic virus distantly related to CCHFV. In addition, Spengler and Estrada-Pena [7] noted that availability of the principal vector of CCHFV and the tick host determines the geographical spread of the virus. Further analysis of our results showed that unmarried herdsmen had higher CCHFV exposure rate compared to the married. Our observation during sampling revealed that unmarried herdsmen had longer contact time (not estimated) with their animals and this increases their exposure risk to tick bites compared to married ones. Also, despite the fact women were not traditionally allowed to graze ruminants, the CCHFV exposure rates were similar in this study. This suggests the existence of other equally competing risk factors among women than during grazing. Generally, young adults (< 36 years), because of their strength, are often observed to be more active in ruminant grazing, and as such, are at higher risk of tick bites and CCHFV exposure than the elderly.

Furthermore, the CCHFV seroprevalence of 91.5% (75/82) among herdsmen who had been bitten by ticks in the course of their work corroborates the role of ticks in the transmission of CCHFV. This study indicated that herdsmen with history of tick bites were 191.3 times more likely to be exposed to CCHFV than those who had no such experience (p<0.0001) (Table 3). More interestingly, the higher significant difference of association in CCHFV seropositivity among febrile patients who had had body contact with ticks and those who had not (p = 0.0132) in this study further highlights exposure to ticks and their bites as a major risk factor in CCHFV transmission.

Comparison of the CCHFV seroprevalence and the risk factors (such as contact with animals, tick bites, tick crushing with bare hands and presence of ticks on clothing) between herdsmen and non-herdsmen febrile patients yielded significantly different (p<0.0001) results. This corroborates previous reports [40, 42] which identified these risk factors as crucial in zoonotic transmission of CCHFV. The non-detection of CCHFV S-segment gene in all the serum samples tested shows that the virus was absent in the samples and suggests that the viraemic phase had passed before sample collection.

Our findings indicate high-level exposure of herdsmen to CCHFV in Kwara State, Nigeria and suggests that the disease is widespread/endemic in the country. In addition, this study provides evidence of silent circulation of CCHFV in this neglected and occupationally at-risk population. The requirements for silent CCHFV circulation such as vector presence, availability and distribution of amplifying hosts, evidence of viral circulation and sharing of international borders with countries having endemic infection cycles as reported by Gordon et al. [10] exist in Nigeria.

In conclusion, considering the potential severity of CCHF in humans coupled with the lack of effective vaccines and therapeutics, nationwide CCHFV surveillance, especially among people in animal-related occupations, domestic animals, and ticks, are needed to fully understand the epidemiology of CCHF in Nigeria. Moreover, improved awareness of risk factors associated with CCHFV infection as well as prevention and control measures are imperative to reduce morbidity and mortality from the disease. Also, CCHF should be considered as a differential diagnosis in cases of febrile illness among humans in the study area.
